# *Dendrobium naungmungense* (Orchidaceae, Dendrobieae), a new species from Kachin State, Myanmar

**DOI:** 10.3897/phytokeys.94.21337

**Published:** 2018-01-29

**Authors:** Qiang Liu, Shi-Shun Zhou, Xiao-Hua Jin, Bo Pan, Kyaw Win Maung, Myint Zyaw, Ren Li, Rui-Chang Quan, Yun-Hong Tan

**Affiliations:** 1 Southeast Asia Biodiversity Research Institute, Chinese Academy of Sciences, Yezin, Nay Pyi Taw 05282, Myanmar; 2 Xishuangbanna Tropical Botanical Garden, Chinese Academy of Sciences, Mengla, Yunnan 666303, China; 3 State Key Laboratory of Systematic and Evolutionary Botany, Institute of Botany, Chinese Academy of Sciences, Beijing 100093, China; 4 HponkanRazi Wildlife Sanctuary Offices, Putao, Myanmar; 5 Forest Research Institute, Forest Department Ministry of Environmental Conservation and Forestry, Yezin, Nay Pyi Taw, Myanmar

**Keywords:** Taxonomy, risk-of-extinction assessment, Khakaborazi National Park

## Abstract

*Dendrobium naungmungense*, a new species from Naungmung, Kachin State, North Myanmar, is described and illustrated. It is morphologically similar to *D. ciliatilabellum* and *D. vexabile*, but the epichile is oblong with three long-ciliate laminae and the column wing has significant denticulation. A preliminary risk-of-extinction assessment shows that the new species should be regarded as Critically Endangered (CR) according to the IUCN Red List Categories and Criteria.

## Introduction

The orchid flora of Myanmar is highly diverse but poorly known, as a result of the past political isolation and instability of the country. The remoteness of many orchid-rich areas and the difficulties of investigation in rugged terrain have also played a role (Ormerod and Kumar 2003; Kurzweil and Lwin 2014). According to recent estimates, about 800 orchid species are distributed in Myanmar (Kurzweil and Lwin 2014), but this is probably an underestimate. Many new distribution records and new species have been published in the last few years (Ormerod 2002, 2012; Ormerod and Kumar 2008; Ormerod and Wood 2010; Nyunt 2006; Kurzweil and Lwin 2012a, b; Tanaka et al. 2011; Tan et al. 2015; Liu et al. 2017; Aung et al. 2017; Yang et al. 2017).

*Dendrobium* Swartz (1799: 82) (Orchidaceae: Epidendroideae; Dendrobieae) is one of the largest genera of Orchidaceae, with approximately 800–1500 species, which are mainly distributed in diverse habitats in South, East and South-east Asia and Australasia, including the Philippines, Borneo, Australia, New Guinea and New Zealand (Cribb and Govaerts 2005; Wood 2006; Zhu et al. 2009). Around 129 species of *Dendrobium* have been recorded from Myanmar (Kurzweil and Lwin 2014; Govaerts et al. 2017). During fieldwork in Khakaborazi National Park, Kachin State, Northern Myanmar since 2015, one new species of *Dendrobium* has been discovered, which is described below. The new species belongs to Dendrobium section Dendrobium Lindl. (Lindley 1844).

## Materials and method

Morphological observations of the new species were carried out based on living plants (five individuals) and dried herbarium specimens (three specimens kept in the herbaria of HITBC and YAF). Measurements were made using a vernier caliper and the descriptive terminology follows Stearn (1983). Both herbarium and fresh specimens of *Dendrobium vexibile* (Liu et al. 2015) were examined under a stereo dissecting microscope. The conservation status of the new species was evaluated based on the guidelines of the International Union for Conservation of Nature (IUCN 2017).

## Taxonomic treatment

### Dendrobium naungmungense

Taxon classificationPlantaeAsparagalesOrchidaceae

Q.Liu & X.H.Jin sp. nov.

urn:lsid:ipni.org:names:77175481-1

Figs 1 , 2

#### Diagnosis.

*Dendrobium naungmungense* is similar to *D. vexabile* and *D. ciliatilabellum*, but can be distinguished by the oblong epichile with three long-ciliate laminae and the margin crisped with hairs and the margin of column wing with significant denticulation.

#### Type.

MYANMAR. Kachin State. Putao County, Naungmung Town, tropical forest, 500–600 m a.s.l., epiphytic on the trunk of riparian trees, 8 Apr 2017, Qiang Liu, *430* (Holotype, HITBC!).

#### Description.

Plant epiphytic, pendent, 30–50 cm long. Stems slender, branching from nodes, internodes covered by sheaths, yellowish, 1.4–2.8 cm long. Leaves anguste-ovate, acute to acuminate, entire, sessile, distichous, 3.0–4.2 × 4.0–5.5 mm. Inflorescence leaf-opposed, 1–2 flowered; peduncle short, sheathed at base, 0.7–0.9 cm long; sheaths membranous, overlapping, 2.5–3.0 mm long; floral bracts broadly lanceolate, three veined membranous, 3.0–4.5 × 2.0–2.5 mm. Flower fragrant, ca. 1.5 cm diameter when open, yellowish green, lip with sparsely purple stripe and spots. Dorsal sepals elliptic, 5-veined, 11.2–12.3 × 6.0–6.5 mm, acuminate; lateral sepals triangular, 5-veined, 12.0–13.5 × 8.0–8.5 mm, apex acuminate; mentum broad, ca. 3 mm. Petals lanceolate, 3-veined, 10.5–11.0 × 4.0–4.5 mm, acuminate. Lip 3-lobed with a short claw, obovate (when spread), 14.5–15.0 × 7.5–8.0 mm, lateral lobes elliptic, 6.0–6.5 × 2.6–3.1 mm, margin with hairs; mid-lobe oblong, 7.5–8.0 × 3.5–3.8 mm, margin crisped with hairs. A broad median band on the hypochile; disc with an ovoid cushion, three long-ciliate laminae extending from base to near apex of epichile. Column ca. 9.0 mm, stelidia falcate with significant denticulation. Operculum subglobose, with densely white crystalline papillate.

**Figure 1.**
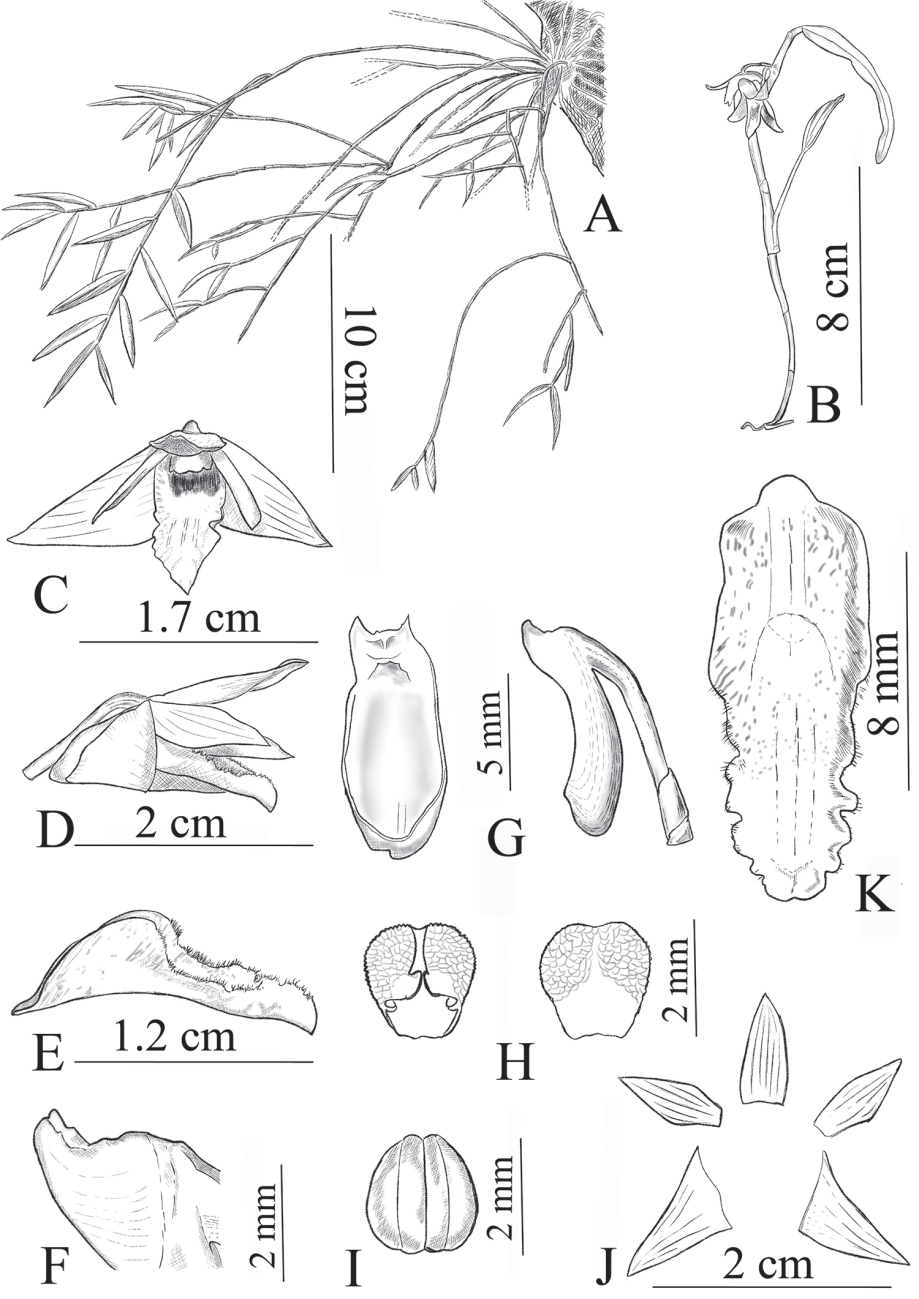
*Dendrobium naungmungense*. **A** Habitat **B** Plant **C** Flower **D** Lateral view of flower **E** Lateral view of labellum **F** Column wing **G** Front and lateral view of column **H** Abaxial and adaxial anther cap **I** Pollinarium **J** Sepals and petals **K** Front view of labellum. All from the type collection (Qiang Liu, *430*) and drawn by Bo Pan.

**Figure 2.**
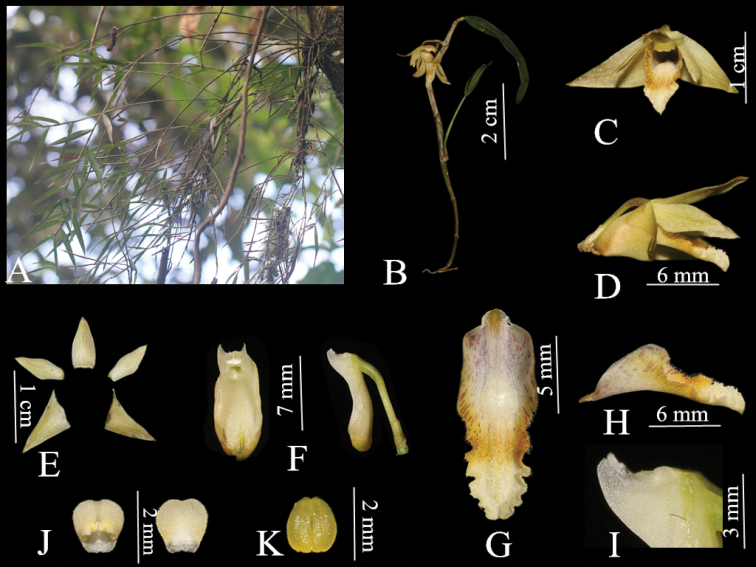
*Dendrobium naungmungense*. **A** Habitat **B** Plant **C** Flower **D** Lateral view of flower **E** Sepals and petals **F** Column **G** Front view of labellum **H** Lateral view of labellum **J** Abaxial and adaxial anther cap **K** Pollinarium **I** Column wing (Photographed by Q. Liu).

**Figure 3.**
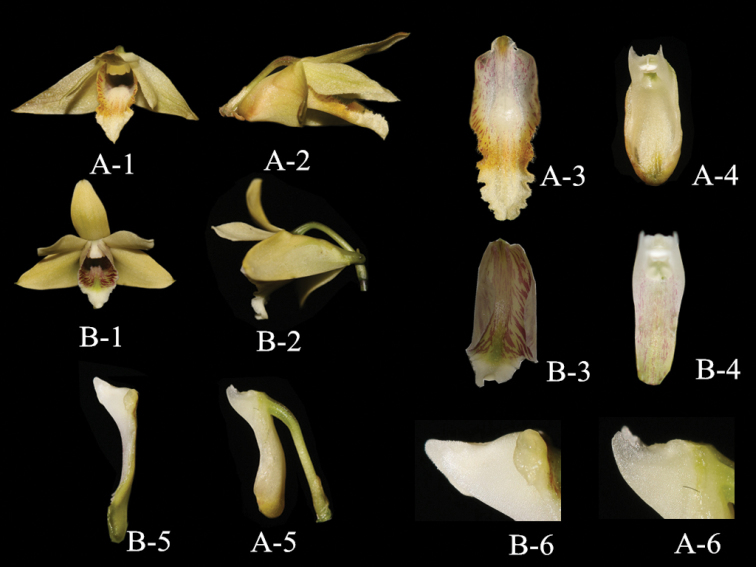
**A** *Dendrobium naungmungense* (**A–1** Front view of flower **A–2** Lateral view of flower **A–3** Labellum **A–4** Front view of column **A–5** Lateral view of column **A–6** Column wing) **B** *Dendrobium vexabile* (**B–1** Front view of flower **B–2** Lateral view of flower **B–3** Labellum **B–4** Front view of column **B–5** Lateral view of column **B–6** Column wing) (Photographed by Q. Liu)

#### Etymology.

The new species is named after Naungmung, Kachin State, North of Myanmar, where it was discovered in a vast area of tropical rainforest.

#### Distribution and habitat.

*Dendrobium naungmungense* is only known from the type locality. It is epiphytic on the trunk of riparian trees in tropical rainforest, which is dominated by *Dipterocarpus obtusifolius* Teijsm. ex Miq. (Dipterocarpaceae).

#### Additional specimens examined (Paratype).

MYANMAR. Kachin State. Putao County, Naungmung Town, tropical forest, 500–600 m a.s.l., epiphyte on the trunk of riparian trees, 11 Jun 2017, Hong Jiang & Qiang Liu, *17017* (YAF!) and 8 Apr 2017, Qiang Liu, *430* (HITBC!).

#### Conservation status.

*Dendrobium naungmungense* was collected in Naungmung Town, Kachin State, Northern Myanmar. Until now, only one population, consisting of ca. 20 mature individuals, has been discovered in 3 years of continuous field investigations (2015–2017). In addition, population growth and human activities have led to serious habitat destruction and deforestation in this region. It is also illegally collected by local people due to the highly ornamental and medicinal values. Hence, according to IUCN Red List Categories and Criteria (IUCN Standards and Petitions Subcommittee 2017), *D. naungmungense* should be regarded as Critically Endangered (CR B1ab (iii, v) + 2ab (iii, v); D), which is the category for species facing the highest risk of extinction in the wild. It meets the IUCN criteria in having an extent of occurrence < 100 km^2^, an area of occupancy < 10 km^2^, is known from a single location and with a continuing decline inferred from the number of mature individuals, a continuing decline in the quality of habitats and a population size of less than 50 mature individuals.

##### Key to *D. naungmungense*, *D. vexabile* and *D. ciliatilabellum*

**Table:** 

1	Mentum (1 × 3 mm), epichile (7.5–8.0 mm) longer than hypochile (6.0–6.5 mm), oblong epichile with three ciliate laminae and margin crisped with dense hairs, column wing with significant denticulation	***D. naungmungense***
–	Mentum (2 × 1 mm), epichile (3.5–4 mm) significantly shorter than hypochile (9–10 mm), ovate epichile without ciliate laminae and margin crisped without hairs, column wing without denticulation	**2**
2	Mid-lobe (2 × 2 mm), disc of lip with densely long-ciliate lamina and un-deflexed epichile	***D. ciliatilabellum***
–	Mid-lobe (4 × 5 mm), disc of lip with sparsely ciliate lamina and de-flexed epichile	***D. vexabile***

## Discussion

Morphologically, *D. naungmungense*is is similar to *D. vexabile* and *D. ciliatilabellum*, which are characterised by branched stems, short inflorescences with 1 or 2 flowers, lip 3-lobed with a narrow claw and flowers yellowish green, except the lip with purple streaks or spots. However, the new species differs from *D. vexabile* and *D. ciliatilabellum* by having a wide mentum, small hypochile, oblong epichile with 3 ciliate laminae and margin crisped with dense hairs and column wing with significant denticulation. Meanwhile, *D. ciliatilabellum* differs from *D. vexabile* by having a small mid-lobe, disc of lip with densely long-ciliate laminae and un-reflexed epichile (large mid-lobe, disc of lip with sparsely ciliate lamina and reflexed epichile in *D. vexabile*) (Seidenfaden 1985, Rao and Haridasan 2005; Liu and Gao 2016).

## Supplementary Material

XML Treatment for Dendrobium naungmungense

## References

[B1] Aung YL Jin X Schuiteman A (2017) *Coelogyne putaoensis* (Orchidaceae), a new species from Myanmar. PhytoKeys 82: 27–34. https://doi.org/10.3897/phytokeys.82.13172 10.3897/phytokeys.82.13172 PMC5546387 28794680

[B2] Cribb P Govaerts R (2005) Just how many orchids are there? In: Raynal-Roques A, Roguenant A, Prat D (Eds) Proceedings of the 18^th^ World Orchid Conference. Naturalia, Dijon, France 161–172.

[B3] Govaerts R Bernet P Kratochvil K Gerlach G Carr G Alrich P Pridgeon AM Pfahl J Campacci MA Holland Baptista D Tigges H Shaw J Cribb PJ George A Kreuz K Wood JJ (2017) World Checklist of Orchidaceae The Board of Trustees of the Royal Botanic Gardens, Kew. http://apps.kew.org/wcsp/monocots/ [Accessed 30 July 2017]

[B4] IUCN Standards and Petitions Subcommittee (2017) Guidelines for Using the IUCN Red List Categories and Criteria. Version 13. Prepared by the Standards and Petitions Subcommittee. http://www.iucnredlist.org/documents/RedList Guidelines.pdf [Accessed: 30 Jul, 2017]

[B5] Kurzweil H Lwin S (2014) A Guide to Orchids of Myanmar. Natural History Publications (Borneo), Kota Kinabalu.

[B6] Kurzweil H Lwin S (2012a) First record of *Taeniophyllum* (Orchidaceae) in Myanmar. The Gardens’ Bulletin, Singapore 64: 133–137.

[B7] Kurzweil H Lwin S (2012b) New records in the orchid flora of Myanmar. Thai Forest Bulletin (Botany) 40: 108–113.

[B8] Liu Q Gao JY (2015) New orchids record in the flora of China. Current Science 110(11): 2064–2066.

[B9] Liu Q Zhou SS Li R Zhang MM Zyaw M Lone S Quan RC (2017) *Bulbophyllum putaoensis* (Orchidaceae: Epidendroideae; Malaxideae), a new species from Kachin State, Myanmar. Phytotaxa 305(1): 57–60. https://doi.org/10.11646/phytotaxa.305.1.9

[B10] Lindley J (1844) Dendrobium. Edwards’ Botanical Register 30: 55.

[B11] Ormerod P (2002) Taxonomic changes in *Gooderinae* (Orchidaceae: Orchidoideae). Lindleyana 17: 189–238.

[B12] Ormerod P Kumar SC (2003) Orchidaceous additions to the flora of Burma (Myanmar). Rheedea 13: 43–50.

[B13] Ormerod P Kumar SC (2008) Orchidaceous additions to the flora of Myanmar 2. Rheedea 18: 75–80.

[B14] Ormerod P Wood EW (2010) A new species of *Pinalia* (Orchidaceae: Eriinae) from Myanmar. Harvard Papers in Botany 15: 349–351. https://doi.org/10.3100/025.015.0215

[B15] Ormerod P (2012) Orchidaceous additions to the floras of China and Myanmar. Taiwania 57: 117–126.

[B16] Rao AN Haridasan K (2005) *Dendrobium vexabile* Rchib. f. (Orchidaceae), a new record to India from Arunachal Pradesh. Arunachal Forest News 21(1/2): 14–16.

[B17] Seidenfaden G (1985) Orchid genera in Thailand XII. Dendrobium Sw. Opera Botanica 83: 153–71.

[B18] Stearn WT (1983) Botanical Latin: History, grammar, syntax, terminology and vocabulary (3^rd^ edn). David & Charles, Newton Abbot, 311–357.

[B19] Swartz O (1799) Dianome epidendri generis Linn. Nova Actaregiae Societatis Scientiarum Upsaliensis, Edman, Upsala 6: 61–88.

[B20] Tanaka N Yukawa T Khin Myo Htwe Koyama T Murata J (2011) New or noteworthy plant collections from Myanmar (7): Fourteen additional species of Orchidaceae. Acta Phytotaxonomica et Geobotanica 61: 161–165.

[B21] Tan Y-H Yang B Li J-W Zhou S-S Lone S Khaing KK Li R Huang J-P Sun H (2015) Acranthera burmanica, a new species of Rubiaceae from Kachin State, Myanmar. Phytotaxa 238(1): 92–96. https://doi.org/10.11646/phytotaxa.238.1.5

[B22] Yang B Zhou SS Maung KW Tan YH (2017) *Reinwardtia glandulifera* (Linaceae), a new species from Kachin State, northern Myanmar. Phytotaxa 316(3): 297–300. https://doi.org/10.11646/phytotaxa.316.3.10

[B23] Wood HP (2006) The *Dendrobiums*. A.R.G, Gantner Verlag, Ruggell, Liechtenstein.

[B24] Zhu GH Tsi ZH Wood JJ Wood HP (2009) *Dendrobium*. In: Wu CY Raven PH Hong DY (Eds) Flora of China (vol. 25). Science Press, Beijing and Missouri Botanical Garden Press, St. Louis, 367–397.

